# New strategies for the treatment of tuberculosis: research progress and clinical translation in immune modulation and inflammation control

**DOI:** 10.3389/fimmu.2026.1974232

**Published:** 2026-10-14

**Authors:** Shihao Du, Lulu Zhen, Ji Zhou, Di Deng

**Affiliations:** 1 Department of Pharmacy, Qingdao Public Health Clinical Center, Qingdao, Shandong, China; 2 Department of Pharmacy, Qingdao Central Hospital, University of Health and Rehabilitation Sciences (Qingdao Central Hospital), Qingdao, Shandong, China; 3 The Prison of Shandong Province, Jinan, Shandong, China

**Keywords:** host-directed therapy, immune training, immunometabolism, immunomodulation, inflammation, tuberculosis

## Abstract

Tuberculosis (TB) remains a major global public health challenge, with treatment complicated by drug resistance, adverse effects, and suboptimal host immune responses. Whilst conventional anti-TB drugs are effective bactericidally, their immunomodulatory effects may, in certain contexts, contribute to immune imbalance and affect treatment outcomes. Host-directed therapy (HDT), which aims to modulate the host immune response to enhance pathogen control and limit immunopathology, has emerged as a promising adjunctive strategy. This narrative review critically evaluates the current evidence for HDT in TB, focusing on immunosenescence, trained immunity, cytokine and lipid mediator networks, immunometabolic reprogramming, neuroendocrine regulation, and repurposed drugs including metformin and statins. Importantly, we distinguish between evidence derived directly from TB models (*in vitro*, animal, and clinical studies) and evidence extrapolated from other diseases, highlighting knowledge gaps and areas requiring further investigation. We propose that future progress depends on evidence-based stratification of HDT candidates, rigorous clinical trial design, and a personalised approach that accounts for patient comorbidities and immune status. This review aims to provide a balanced framework for advancing HDT from bench to bedside in TB therapy.

## 1 Introduction

Treatment for tuberculosis (TB) has begun to move away from a single bactericidal approach and has changed. Although Directly Observed Short-course Chemotherapy (DOTS) is effective, its extended period of use, serious toxic side effects, and possible immunosuppression of the host immune system together lead to poor patient adherence to treatment, treatment failure, recurrence, and the emergence of drug-resistant strains (1). In severe tuberculosis, the main cause of pathological changes is an excessive immune response generated by the host to control bacterial proliferation (2). Sustained inflammation caused by pro-inflammatory factors in the host, a result of a large quantity of bacteria, leads to damage in the lungs, such as lung cavities and fibrosis (2). Therefore, the demand to improve clinical outcomes and reduce the duration of treatment has prompted the development of a two-pronged strategy: new antibacterial drugs and host-directed therapy HDT to modulate the immune system’s response to *Mycobacterium tuberculosis* (Mtb) (2). In recent years, the HDT strategy of regulating the host immune response and reducing pathological inflammation has become a new area of research in tuberculosis and is a promising direction for adjuvant therapy (1).

HDT aims to strengthen the entire regulation of Mtb by modifying the host’s immune microenvironment to enhance the host’s own anti-tuberculosis defence mechanism and reduce tissue damage caused by an overly strong immune response; it does not directly kill bacteria (1). HDT strategies are immunomodulatory to reduce non-productive inflammation and enhance antimicrobial activity; therefore, they can protect the lungs from damage and improve symptoms (2). For instance, in pulmonary tuberculosis, excessive inflammation may cause damage to the alveolar tissue and lead to cavitation; such inflammation is even more severe in patients with comorbid tuberculosis and diabetes (3). Therefore, an attenuation of the inflammatory response is to be expected in HDT; at the same time, it must not be too strong, or the immune system will be unable to fight against Mtb and thus allow bacteria to multiply more freely (3). Further dysregulation of the type I interferon system and host cell metabolism may also occur under tuberculosis-diabetes co-morbidity and lead to a poor prognosis; thus, a moderate-level regulation of both inflammation and the immune system should be achieved (3).

This narrative review critically evaluates current mechanistic insights, promising strategies, and translational challenges in immune modulation and inflammation control for pulmonary TB. We conducted a focused literature search in PubMed (2015–2026) using keywords including “tuberculosis,” “host-directed therapy,” “immunomodulation,” “inflammation,” and “repurposed drugs.” Unlike previous reviews, our primary objective is to critically assess the strength of evidence for each HDT candidate, explicitly distinguishing between: (1) direct evidence from TB-specific studies; (2) indirect evidence extrapolated from other diseases; and (3) purely hypothetical mechanisms.

Evidence Classification Framework. To enhance scientific rigor, we adopt the following evidence hierarchy: Level A (RCTs in TB patients); Level B (observational studies in TB patients); Level C (preclinical TB animal models); Level D (*in vitro* TB studies); Level E (evidence extrapolated from non-TB diseases); Level F (hypothetical mechanisms). This framework is applied in tables and throughout the text.

## 2 Immunological ageing and immune training: a new perspective on the treatment of pulmonary tuberculosis in the elderly

### 2.1 The impact of immunological ageing on susceptibility to and treatment of pulmonary tuberculosis

With the increase of the world’s population’s age, many older adults have been facing higher burdens of pulmonary tuberculosis in recent years. Immunological ageing is the main biological reason for the increased risk of tuberculosis in older people, higher risk of reactivation of latent infections, and poor treatment response (4, 5). Studies have shown that most tuberculosis cases in older adults are related to the reactivation of latent foci, and immunological ageing is the main cause of this reactivation (6). A multi-centre study in France has also found that older tuberculosis patients frequently exhibit immunological ageing, their clinical features are often non-typical, and thus diagnosis is delayed and mortality is higher (7). Immunological ageing refers to the decline in function of both the innate and adaptive immune systems with age. At the level of innate immunity, the phagocytic and bactericidal capabilities of first-line defence cells, such as alveolar macrophages, are reduced (8). At the level of adaptive immunity, both the proliferative capacity and effector functions of T cells are impaired, resulting in a reduced efficiency of the specific immune response to Mtb (5). More seriously, immunological ageing is associated with a prolonged, low-grade systemic inflammatory state known as ‘inflammaging’, and this inflammatory dysregulation is a characteristic feature of ageing immunity (8). Ageing of the immune system in older adults is associated with impaired immune surveillance against Mtb and reduced clearance of pulmonary tuberculosis lesions (6). At the same time, chronic, low-grade inflammation in older adults can damage lung tissue and affect the effectiveness of anti-tuberculosis treatment (8). For instance, patients with multidrug-resistant tuberculosis (MDR-TB) have shown high levels of oxidative stress and inflammation, which are closely related to ageing and immunosenescence, and thus may affect treatment outcomes (9). Additionally, older adults often have multiple comorbidities, malnutrition, or take other medications; these reasons can increase the risk of adverse drug reactions and reduce treatment compliance, and may also further impair the immune system, creating a vicious cycle (10, 11). Therefore, to improve the clinical outcome of older adults with pulmonary tuberculosis, the exact role of immunosenescence in the development and treatment of this disease in older people must be understood.

### 2.2 Immune training as an immunomodulatory strategy for tuberculosis in the elderly

Immune training is a type of enhancement in the ability of innate immune cells to respond to following infections caused by different pathogens after being exposed once to a specific stimulus (such as vaccines), achieved through epigenetic modification and metabolic change. In the context of tuberculosis in older adults, the activation of trained immunity has been proposed as a new type of HDT. The first aim is to counteract the age-related decline in adaptive immunity by strengthening the antibacterial and immunomodulatory functions of innate immune cells (11). Research has shown that activators of trained immunity, such as some microbial factors and their metabolic products, can correct the imbalance between the innate and adaptive immune systems in older adults (11). Rebalancing can help reduce the excessive inflammatory response of ‘inflammatory ageing’ and thus alleviate immunopathological damage; at the same time, it can strengthen the innate antibacterial ability of Mtb and control the infection more effectively (11). For example, the cytokine interleukin-27 (IL-27) has been shown to induce autophagy in human serum-induced macrophage differentiation through a non-canonical path that is independent of mammalian target of rapamycin (mTOR) and microtubule-associated protein light chain 3 (LC3) (12). Autophagy is a necessary pathway for macrophages to clear intracellular pathogens; therefore, factors that regulate the function of innate immune cells, such as IL-27, may share common ground in their mechanisms with the idea of immune training and provide new directions for the development of immunomodulators. However, whilst IL-27 induces an important antimicrobial response, it is currently hypothetical whether this represents a form of trained immunity, which is defined by persistent epigenetic and metabolic reprogramming. The distinction between acute immunomodulation and bona fide trained immunity warrants further investigation. Epigenetic reprogramming can reverse or ameliorate age-related immune dysfunction and offer new approaches to personalised treatment of tuberculosis in older people (11). Although the Bacillus Calmette-Guérin (BCG) vaccine is an older type of tuberculosis vaccine, its protective effect may be weakened by immunosenescence, and it is contraindicated for people with HIV; thus, there is a need to develop new immunological intervention strategies specifically for older adults (13). In the future, more research will be conducted to explore in detail the specific molecular mechanisms and physiological effects of immune activators in elderly people to design highly effective tuberculosis prevention and treatment plans for older adults (**Figure 1**; **Table 1**).

**Figure 1:**
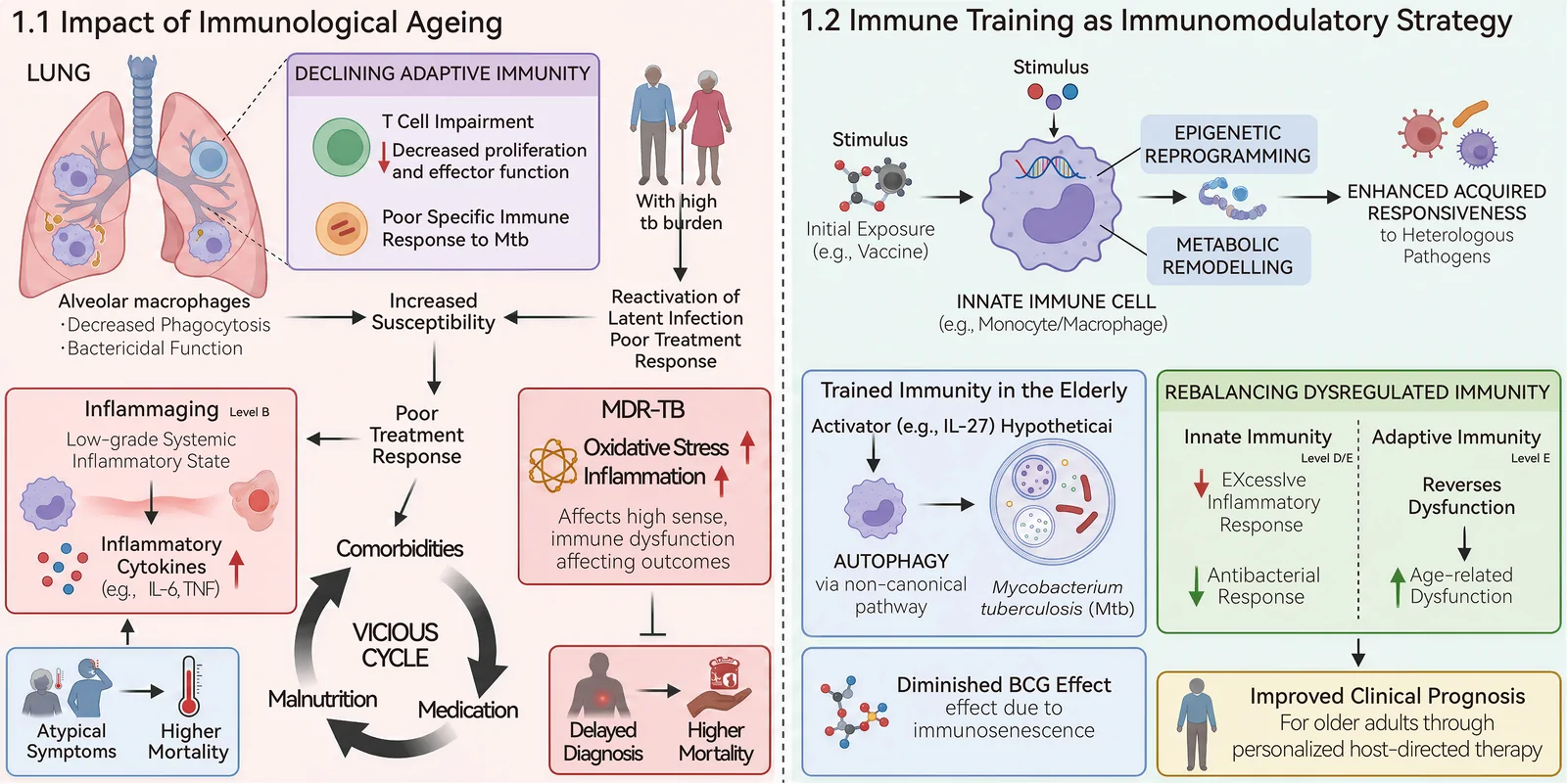
Immunological ageing and trained-immunity-based immunomodulatory strategies in elderly tuberculosis. Left Consequences of immunological ageing for tuberculosis. Advanced age leads to dysfunction of alveolar macrophages and impaired T-cell adaptive immunity, raising susceptibility to Mycobacterium tuberculosis (Mtb) infection and latent TB reactivation. Low-grade chronic inflammation (inflammaging) fuels a vicious cycle together with comorbidities, malnutrition and medication, resulting in atypical clinical manifestations, delayed diagnosis, higher mortality and poor treatment responses amongst elderly TB patients. Oxidative stress and excessive inflammation further worsen outcomes of multidrug-resistant-TB (MDR-TB). 1.2 Immune training as an immunomodulatory strategy. Upon initial stimuli such as vaccination, innate immune cells undergo epigenetic reprogramming and metabolic remodelling to establish trained immunity, enhancing responsiveness against heterologous pathogens. In the elderly, immunosenescence weakens BCG-induced trained immunity; activators such as IL-27 can trigger autophagy-dependent anti-mycobacterial responses. Personalised host-directed therapy rebalances dysregulated innate and adaptive immunity and improves clinical prognosis for older TB adults. Evidence levels: A, RCTs in TB patients; B, observational studies in TB patients; C, preclinical TB animal models; D, *in vitro* TB studies; E, evidence extrapolated from non-TB diseases; F, hypothetical mechanisms.

**Table 1:** Key targets and intervention strategies related to immunosenescence and trained immunity in elderly pulmonary tuberculosis.

Category	Key molecules/cells	Immune alteration in elderly TB	Candidate intervention	Expected outcome	Evidence level
Innate immunity	Alveolar macrophage	Reduced phagocytic/bactericidal capacity	Trained-immunity activators (IL-27, microbial agonists)	Restore autophagy; enhance mycobacterial clearance	Level D (*in vitro*) (12); Level F (trained immunity hypothesis)
Adaptive immunity	T lymphocyte	Impaired proliferation/effector function	Epigenetic reprogramming agents	Recover Th1 responses	Level E (non-TB mechanistic) (11)
Chronic low-grade inflammation	TNF-α, IL-6	Persistent systemic inflammation	Anti-inflammatory HDT adjuvants	Mitigate immunopathological lung injury	Level B (observational/review in elderly TB) (8)
Trained-immunity-related	BCG, IL-27	BCG-induced trained immunity blunted by immunosenescence	Optimised trained-immunity immunomodulators	Rebalance innate-adaptive crosstalk; lower reactivation risk	Level C/D (TB animal/*in vitro*) (47); Level F (elderly TB)

This table summarises the main immune alterations caused by immunosenescence in elderly TB patients and candidate trained-immunity-based host-directed therapy interventions. Evidence levels: A, RCTs in TB patients; B, observational studies in TB patients; C, preclinical TB animal models; D, *in vitro* TB studies; E, evidence extrapolated from non-TB diseases; F, hypothetical mechanisms. References are numbered according to the main reference list. Abbreviations: BCG, Bacillus Calmette-Guérin; HDT, host-directed therapy; IL, interleukin; Mtb, Mycobacterium tuberculosis; TB, tuberculosis; TNF-α, tumor necrosis factor-alpha.

## 3 Network regulation of cytokines and lipid mediators

### 3.1 The pathological role of cytokine storms and immune dysregulation

The concept of a “cytokine storm”—characterised by excessive release of pro-inflammatory cytokines (e.g., TNF-α, IL-1β, IL-6, IL-17, IFN-γ) alongside insufficient anti-inflammatory regulation (e.g., IL-10)—is a central feature of severe TB and is exemplified in TB-COVID-19 co-infection (14–16). This immune imbalance fails to control the pathogen and drives tissue damage, including pulmonary necrosis and cavitation (16). Whilst TB-COVID-19 co-infection provides an instructive model of cytokine dysregulation, the core principle—restoring immune balance—is broadly applicable to HDT in TB (17–19). Therefore, we focus the following discussion on HDT strategies targeting cytokine networks, rather than on co-infection-specific features.In severe TB-COVID-19 co-infection, systemic upregulation of S100 family proteins, mainly released by classical monocytes, contributes to the inflammatory cytokine storm via the S100-TLR4-MyD88 signalling pathway (15).

### 3.2 Eicosanoids and oxylipins: an emerging frontier and hypothetical pathway for inflammation regulation

Eicosanoids are a group of lipid mediators produced through the oxidation of polyunsaturated fatty acids, and although they also regulate inflammation to the same extent as the cytokine network, they have been studied much less. They form an intricate lipid signalling network that cooperates with cytokines to regulate the start, expansion and termination of the immune response precisely. Currently, emerging evidence suggests that Mtb manipulates the balance of oxylipins to modulate host inflammatory responses and escape immune detection. However, direct evidence for oxylipin-targeted HDT in TB remains limited. The following discussion is therefore hypothesis-generating, based on pathways established in other inflammatory diseases (20, 21). For example, in severe COVID-19, an exaggerated inflammatory response is associated with cytokine storms, and metabolic disturbances in lipid mediators may also be involved in this process (20). To explore new pathways of immune regulation, scientists have begun to investigate characteristic lipid mediator profiles produced by the host in response to various pathogen stimuli, known as ‘oxylipin signatures’. For instance, significant differences exist in the lipid metabolic response of the host under bacterial stimulation, such as lipopolysaccharide (LPS) or BCG, compared with that induced by viral stimuli (21). These distinct oxylipin response patterns, together with specific cytokine profiles, are collectively regarded as features of immune recognition and response to various pathogens (21, 22). Targeting the synthesis or signalling pathways of these specific lipid mediators has been proposed as a new direction for the development of HDT that does not rely on traditional cytokine targeting (22). We propose that the “oxylipin signature” could represent a novel dimension for HDT, for example, by balancing pro-inflammatory lipid mediators (e.g., leukotrienes) against pro-resolving mediators (e.g., lipoxins, protectins) to limit excessive inflammation and promote tissue repair (22). However, this remains a speculative framework requiring dedicated investigation in TB-specific models and patient cohorts (**Figure 2**).

**Figure 2:**
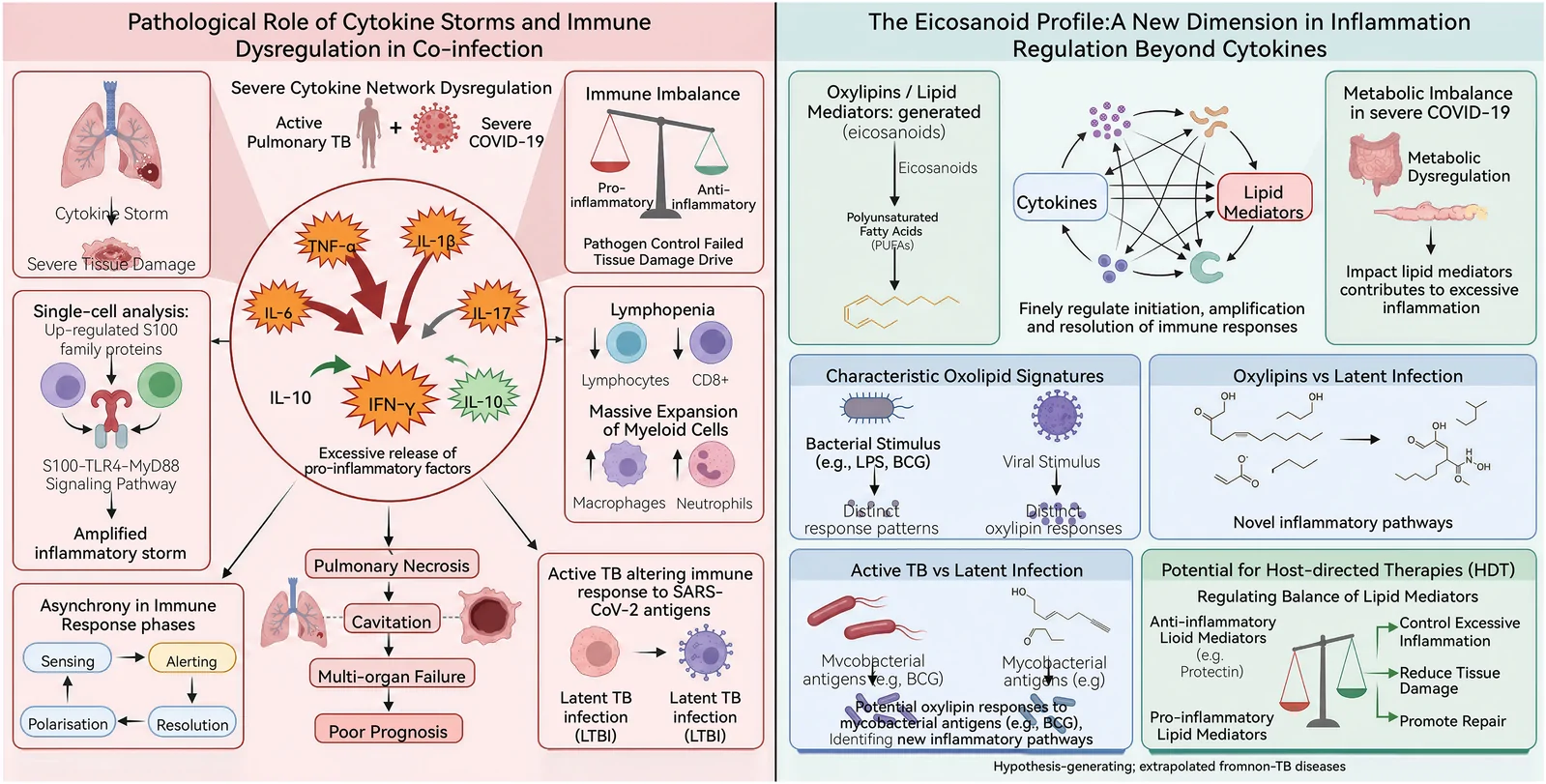
Cytokine-mediated immunopathology and eicosanoid-based lipid regulation in tuberculosis-related co-infection. Left Pathological mechanisms of cytokine storm and immune dysregulation during TB-COVID-19 co-infection. Active pulmonary TB combined with SARS-CoV-2 infection provokes excessive release of pro-inflammatory cytokines including TNF-α, IL-1β, IL-6, IL-17, and IFN-γ. Up-regulated S100-family proteins activate the S100-TLR4-MyD88 signalling axis and further amplify inflammatory cascades. Immune imbalance, lymphopenia and myeloid-cell expansion result in pulmonary necrosis, cavitation and multi-organ failure, ultimately leading to poor prognosis. Latent tuberculosis infection can also alter host immune responsiveness to SARS-CoV-2 antigens. Right Eicosanoid-oxylipin lipid mediators as an additional layer for inflammatory regulation beyond cytokines. Eicosanoids derived from polyunsaturated fatty acids cooperate with cytokines to orchestrate the full course of immune responses. Bacterial and viral stimuli trigger distinct oxylipin signatures. Metabolic disturbance during severe infection reshapes lipid-mediated inflammation, and differential oxylipin responses exist between active and latent TB. Balancing pro-inflammatory and anti-inflammatory lipid mediators provides new perspectives for host-directed therapy to limit excessive inflammation, mitigate tissue injury and promote tissue repair. Evidence levels: A, RCTs in TB patients; B, observational studies in TB patients; C, preclinical TB animal models; D, *in vitro* TB studies; E, evidence extrapolated from non-TB diseases; F, hypothetical mechanisms.

## 4 The cross-talk between metabolic reprogramming and immune regulation

### 4.1 Metabolic switching in immune cells and tuberculosis outcomes

Mtb infection significantly alters the metabolic pathways in host immune cells to create an intracellular environment favourable for the proliferation of Mtb. As the first line of defence against Mtb, macrophages significantly change their metabolic programmes during infection, and thus alterations in glucose metabolism have attracted considerable attention (23). These modifications include changes in glycolysis, the pentose phosphate pathway and the tricarboxylic acid cycle, all of which aim to meet the high-energy demand imposed by the pathogen. Research has shown that Mtb infection induces a shift in macrophages to fatty acid oxidation; this is a relatively quiescent metabolic state that can help maintain the bacteria (24). Alterations in the metabolic status affect both the energy supply for macrophages and their activation status, polarisation and function. For example, the metabolic switch to glycolysis is closely associated with macrophage polarisation into the pro-inflammatory (M1) type and enhanced antibacterial activity (23). Metabolism and the immune system are closely related, so they have been referred to as immunometabolism; thus, a new path for investigating the impact of tuberculosis has been proposed. Mitochondria are multifunctional components of both energy production and the innate immune system, and they produce various types of mitochondrial reactive oxygen species (mtROS) that act as numerous signalling factors connecting mitochondrial metabolism to the ensuing innate immune response (25). Following Mtb infection, alterations in the quantity of mtROS can affect all parts of inflammation, lipid peroxidation and cell death (25). Therefore, by targeting immunometabolism, one can inhibit specific metabolic enzymes induced by Mtb or use drugs that enhance glycolysis to gradually emerge a host-directed therapy strategy for regulating the direction of inflammation and boosting bacterial clearance (24). For example, Metformin maintains metabolic homeostasis in immune cells to increase the host’s innate resistance to Mtb infection in active tuberculosis and further demonstrate the therapeutic value of metabolic modulation (26). Metabolic heterogeneity of immune cells is also closely associated with the microenvironment of tuberculous granulomas, and together they determine the final outcome of the infection (**Figure 3**) (27).

**Figure 3:**
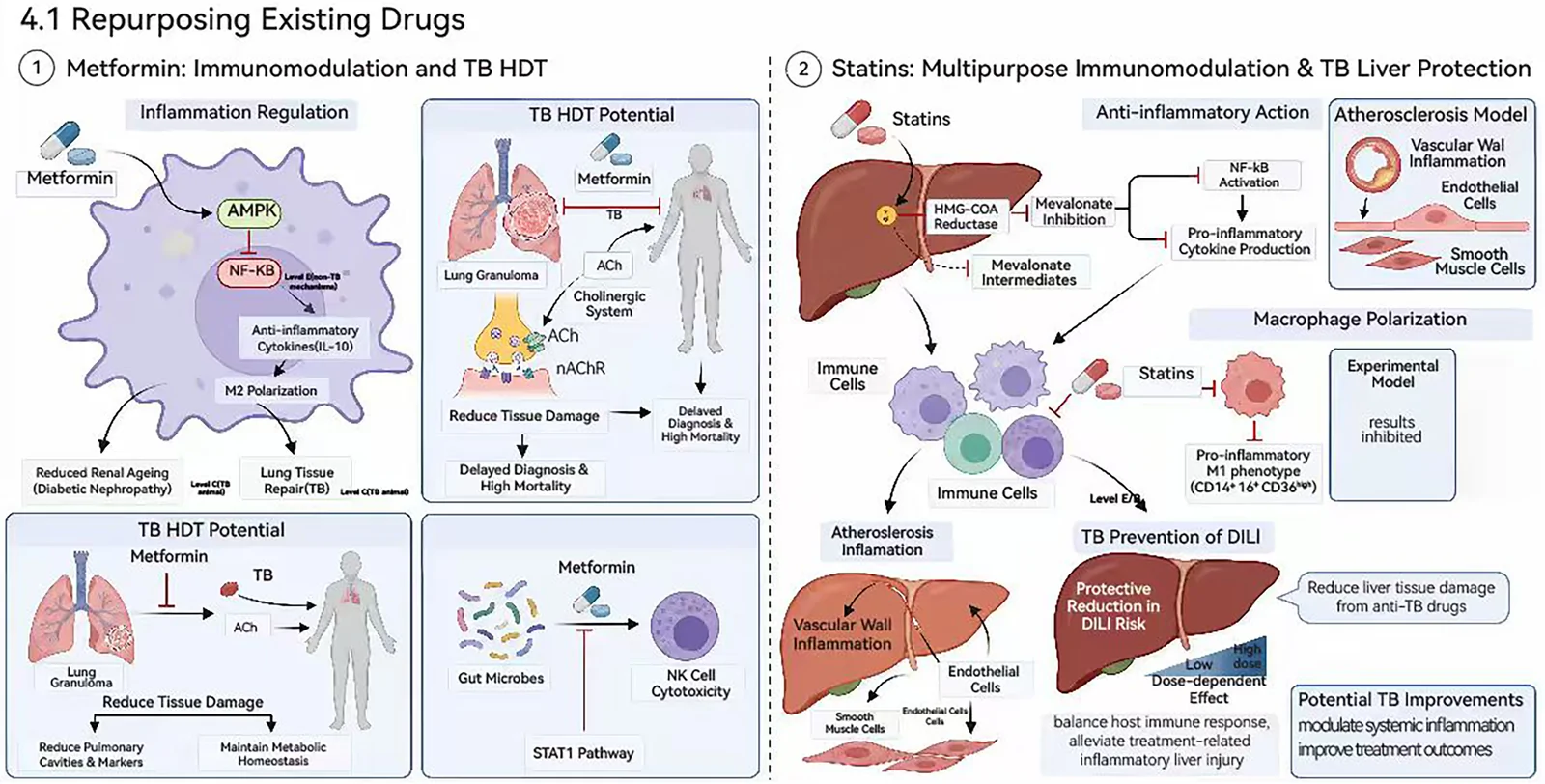
Repurposed metformin and statins as host-directed therapy for tuberculosis. Left Immunomodulatory mechanisms of metformin in tuberculosis host-directed therapy. Metformin activates AMPK and inhibits NF-κB signalling to shift cytokine balance towards anti-inflammatory profiles and promote M2 macrophage polarisation, facilitating lung tissue repair. It ameliorates tissue injury within lung granulomas through cholinergic-system modulation and maintains metabolic homeostasis. Metformin also reshapes gut microbiota to enhance NK-cell cytotoxicity via the STAT1 pathway, conferring protective benefits for tuberculosis patients. Right Multifaceted immunomodulation and liver-protective effects of statins for tuberculosis. Statins suppress HMG-CoA reductase-dependent mevalonate synthesis, restraining NF-κB-mediated pro-inflammatory cascades and modulating macrophage polarisation. Beyond anti-atherosclerotic vascular protection, statins exert dose-dependent mitigation against anti-tuberculosis-drug-induced liver injury (DILI). Repurposed statins balance host immune responses and alleviate treatment-related hepatic damage, improving overall tuberculosis treatment outcomes. Evidence levels: A, RCTs in TB patients; B, observational studies in TB patients; C, preclinical TB animal models; D, *in vitro* TB studies; E, evidence extrapolated from non-TB diseases; F, hypothetical mechanisms.

### 4.2 The cholinergic system: a preclinical target with translational uncertainties

Recently, research has shown that the cholinergic system is involved in the immunometabolic regulation of tuberculosis, and its way of working is rather complicated and closely linked to the course of the disease. Studies have discovered that Mtb can produce acetylcholine (ACh) and increases the expression of non-neuronal cholinergic systems in the lungs during infection, especially in severe, progressive tuberculosis (28). The levels of choline acetyltransferase (ChAT) and nicotinic acetylcholine receptors (nAChRs, such as the α7 subunit) in pulmonary epithelial cells and immune cells are elevated during infection. ACh exerts an anti-inflammatory effect through nAChRs, and thus may create a microenvironment favourable to bacterial growth and disease development (28). Preclinical evidence suggests that pharmacological intervention at this level may be therapeutic. In a TB animal model, nAChR antagonists increased pro-inflammatory cytokine production, reduced bacterial load, and showed synergy with antibiotics (28). Whilst this raises the intriguing possibility of a new therapeutic target—even for multidrug-resistant TB—these findings are currently restricted to experimental settings, and their clinical translatability remains to be established (28). Cholinergic system activation is a type of immune evasion by Mtb that suppresses the host’s excessive inflammatory response to help the bacteria survive. Preclinical evidence suggests a therapeutic effect in experiments. For example, in an animal model of TB, nAChR antagonists increased the production of pro-inflammatory cytokines and reduced the bacterial load; they were also synergistic with antibiotics (28). The Regulatory Function of the Cholinergic System is Linked to the Metabolic Status of Immune Cells. For example, the metabolic reprogramming induced by Mtb infection alters the function of immune cells, and ACh signalling may further modify these metabolic pathways. Research has shown that the metabolic status of host immune cells and the ratio of glycolysis to oxidative phosphorylation are required for pathogen clearance (29). Overexpression of cholinergic anti-inflammatory pathways may inhibit the required pro-inflammatory metabolic shift, thus suppressing the antibacterial immune response. Therefore, antagonism of nAChR signalling could alleviate this inhibition and enable immune cells to resume an effective pro-inflammatory and antibacterial metabolic programme, such as enhanced glycolysis and the generation of related antibacterial effector molecules (23).

Importantly, a critical trade-off must be acknowledged: enhancing pro-inflammatory responses may improve bacterial control but could also exacerbate immunopathological lung damage. This balance between antimicrobial efficacy and tissue protection is a central challenge in HDT development and requires careful evaluation in future studies. Therefore, the cholinergic system remains an experimental target rather than a clinically validated HDT strategy. Provide a theoretical basis for the development of host-directed therapy that simultaneously targets the cholinergic system and immune metabolism; aim to rebalance the host’s immune response for better control of infection; and potentially reduce tissue damage caused by immunopathology (**Figure 4**).

**Figure 4:**
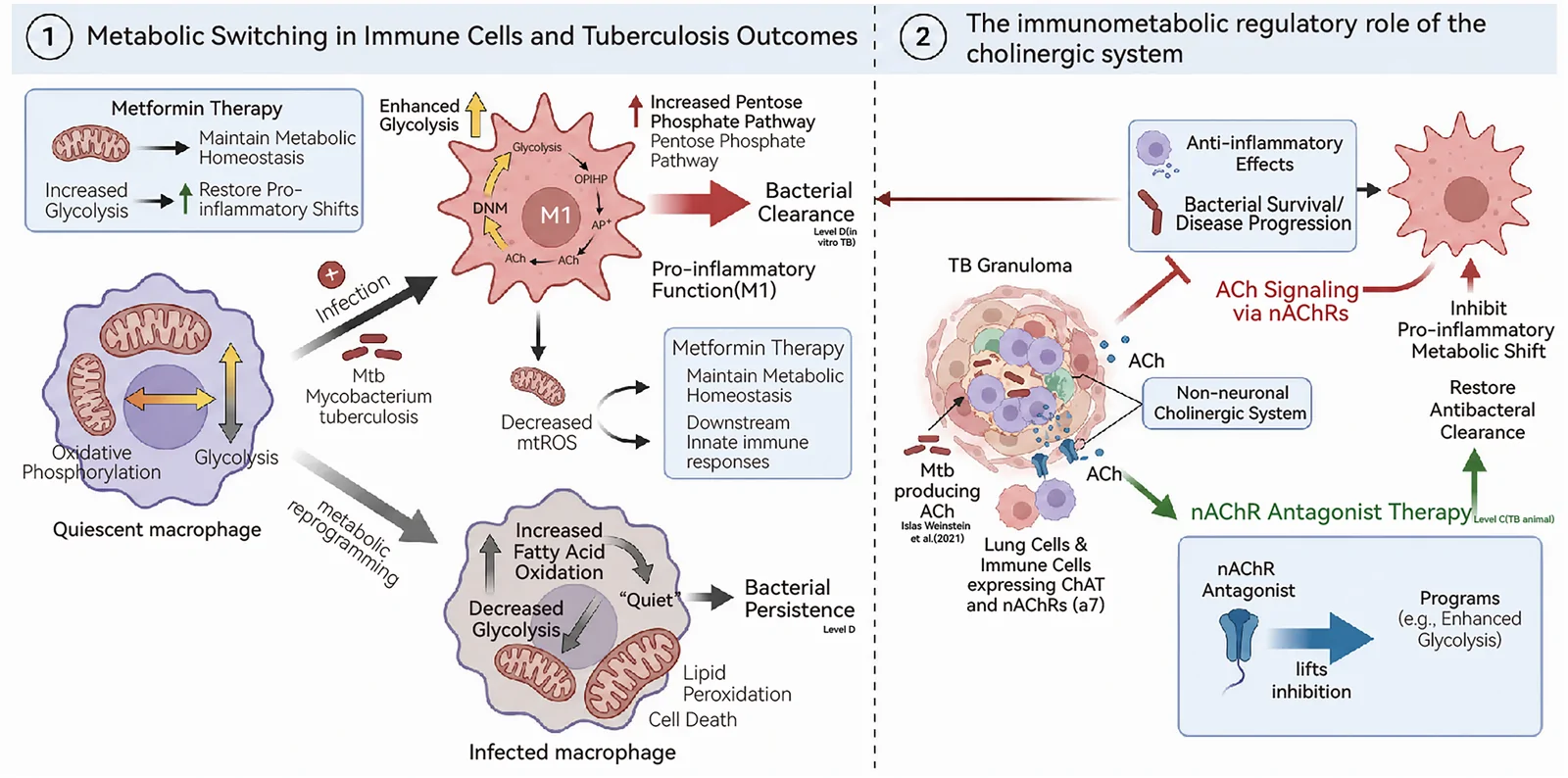
Macrophage immunometabolic switching and cholinergic-immune crosstalk during Mycobacterium tuberculosis infection. (Left) Upon Mycobacterium tuberculosis infection, quiescent macrophages undergo metabolic reprogramming. Enhanced glycolysis drives M1-like pro-inflammatory responses and bacterial clearance, whereas suppressed glycolysis and elevated fatty-acid oxidation promote a “quiet” macrophage state favouring bacterial persistence. Metformin preserves metabolic homeostasis to sustain innate immune function. (Right) Non-neuronal cholinergic signalling within tuberculous granuloma mediates bacterial immune evasion. Acetylcholine (ACh) acting through nicotinic acetylcholine receptors (nAChRs) suppresses protective pro-inflammatory metabolic shifts and promotes disease progression. Pharmacological nAChR antagonism lifts this inhibitory effect, restores antibacterial metabolic programmes, including enhanced glycolysis, and facilitates bacterial clearance. Evidence levels: A, RCTs in TB patients; B, observational studies in TB patients; C, preclinical TB animal models; D, *in vitro* TB studies; E, evidence extrapolated from non-TB diseases; F, hypothetical mechanisms.

## 5 Novel immunomodulators and host-directed therapy candidates

### 5.1 Repurposing of existing drugs: metformin and statins

Repurposed drugs represent a pragmatic avenue for HDT in TB. **Table 2** summarises the primary indications, molecular targets, immunomodulatory effects, evidence levels, and main limitations of the repurposed agents discussed in this section.

**Table 2:** Overview of targets, immunomodulatory effects, evidence levels, and main limitations of repurposed host-directed adjuvant agents against tuberculosis.

Drug	Primary indication	Molecular target	Immunomodulatory effects in TB	Evidence level (in TB Context)	Main limitations
Metformin	Type-2 diabetes	AMPK/NF-κB axis	Promote macrophage autophagy; balance M1/M2; alleviate pulmonary inflammatory injury	Level A (RCT, negative primary endpoint) (36); Level C (TB animal) (26); Level E (non-TB mechanisms) (31–33)	Sputum conversion benefit inconsistent; safety in severe renal impairment
Statins	Dyslipidemia	HMG-CoA reductase	Inhibit pro-inflammatory response; modulate macrophage polarisation; reduce anti-TB DILI	Level B (observational, hepatoprotection) (39); Level E (non-TB immunomodulation) (40–42); Level F (direct TB immunomodulation)	Lack of large-scale RCTs; causality not established
Azithromycin	Bacterial respiratory infections	Neutrophil inflammatory pathway	Suppress excessive neutrophil-mediated inflammation	Level B (pilot RCT) (4)	Long-term bacterial resistance risk

This table summarises repurposed drugs (metformin, statins, azithromycin) evaluated as HDT candidates for TB, including their primary indications, molecular targets, immunomodulatory effects, evidence levels in the TB context, and main limitations. Evidence levels: A, RCTs in TB patients; B, observational studies in TB patients; C, preclinical TB animal models; D, *in vitro* TB studies; E, evidence extrapolated from non-TB diseases; F, hypothetical mechanisms. References are numbered according to the main reference list. Abbreviations: AMPK, AMP-activated protein kinase; DILI, drug-induced liver injury; HMG-CoA, 3-hydroxy-3-methylglutaryl-coenzyme A; NF-κB, nuclear factor kappa B; RCT, randomised controlled trial; TB, tuberculosis.

#### 5.1.1 Metformin: evidence for anti-inflammatory effects but not for bacteriological benefit

Metformin is an oral hypoglycemic agent that has attracted attention for its immunomodulatory properties in various diseases, including cancer, autoimmune disorders, aging, COVID-19, and tuberculosis (30). Mechanistically, metformin activates AMPK and inhibits NF-κB signalling, reducing pro-inflammatory cytokines such as IL-6 and TNF-α (31, 32). In non-TB disease models—such as diabetic nephropathy—metformin reduced IL-6 and TNF-α via the FABP4/FOXO1 axis, increased IL-10, and promoted M2 macrophage polarisation (33). In cancer models, it enhanced NK-cell cytotoxicity through CXCL1 inhibition and STAT1 activation (34), and restored immunoregulatory properties of mesenchymal stem cells via metabolic reprogramming (35). However, these mechanistic insights are derived largely from non-TB models and require validation in TB-specific settings.

The most direct clinical evidence for metformin in TB comes from a randomised controlled trial in which metformin add-on therapy did not improve sputum culture conversion rates at 8 weeks (36). Nevertheless, secondary analyses from the same trial suggested potential benefits in reducing pulmonary cavitary lesions and inflammatory markers (36). This discordance—improved inflammation parameters without accelerated bacteriological clearance—highlights a critical distinction between metformin’s effects on immunopathology versus its effects on microbiological control.

Preclinical studies in guinea pigs have shown that metformin can modify the metabolic status of Mtb-infected immune cells and reduce lung damage in chronic active TB (26). In summary, current evidence does not establish metformin as an effective adjunctive HDT for TB, particularly for improving bacteriological outcomes. However, its potential to modulate inflammation and limit lung pathology warrants further investigation in well-designed, mechanism-based clinical trials with patient stratification. In summary, current evidence does not establish metformin as an effective adjunctive HDT for TB, particularly for improving bacteriological outcomes. However, its potential to modulate inflammation and limit lung pathology warrants further investigation in well-designed, mechanism-based clinical trials with patient stratification (**Table 2**).

#### 5.1.2 Statins: hepatoprotective effects versus direct immunomodulation

##### 5.1.2.1 Evidence for hepatoprotection

Statins are primarily used for lipid lowering, but also possess anti-inflammatory and immunomodulatory properties (37, 38). A retrospective cohort study of 1,312 patients with active TB in Taiwan found that statin use was associated with a significantly lower risk of anti-TB drug-induced liver injury (DILI), with a dose-dependent protective effect (39). This observation, whilst promising, comes from a single retrospective study and cannot establish causality. Potential confounding factors—including baseline liver function, comorbidities, and concomitant medications—must be acknowledged. Importantly, prevention of DILI, whilst clinically valuable, is not equivalent to HDT; hepatoprotection does not necessarily imply modulation of TB-specific immunopathology.

##### 5.1.2.2 Evidence for direct immunomodulation (mostly extrapolated)

Beyond hepatoprotection, statins possess broad anti-inflammatory properties, including inhibition of HMG-CoA reductase, suppression of NF-κB activation, and modulation of macrophage polarisation (40, 42). For example, statins can directly reduce inflammation in atherosclerosis models and inhibit polarisation of human macrophages into a pro-inflammatory phenotype (41, 42). However, the majority of this evidence comes from studies of atherosclerosis, cancer, and other non-TB diseases. Whether statins can directly modulate the host immune response to Mtb in humans remains largely unknown. Whilst their anti-inflammatory profile is theoretically attractive for limiting TB-associated tissue damage, direct evidence in TB models is limited, and large-scale randomised trials are lacking. Therefore, the role of statins as HDT for TB remains experimental and hypothesis-generating, requiring dedicated clinical investigation.

In summary, statins have moderate evidence for hepatoprotection (Level B, observational) but lack direct immunomodulatory evidence in TB (Level E/F for this indication). Their use as HDT for TB is not currently supported by clinical evidence.

## 6 The role of epigenetic and transcriptional regulation in immune modulation

### 6.1 Epigenetic reprogramming, trained immunity and disease discrimination

The fundamental mode of action for trained immunity is modification of the epigenome in innate immune cells to induce an extended enhancement of immune function. Research has shown that trained immunity imparts memory function in innate immune cells through metabolic and epigenetic alterations; such changes include modifications to histones (43). For example, the long non-coding RNA TRCR1 in plasma exosomes from individuals resistant to TB increases CLOCK mRNA levels by binding to the RNA-binding protein FXR2. This increases the expression of the circadian regulator CLOCK and promotes CLOCK-mediated acetylation of histone H3 (K9/K14) at the promoter regions of immune genes, thus conferring antibacterial activity through epigenetic memory (43). This reprogramming does not occur only in circulating immune cells but also in immune cells within the lungs. Genome-wide DNA methylation analysis of alveolar macrophages and alveolar T cells in individuals exposed to Mtb showed that the DNA methylation profiles of these cells were significantly different from those in unexposed individuals; furthermore, they formed distinct clusters, and the differentially methylated genes were functionally related to trained immunity and tuberculosis (44). Additionally, the status of the interferon-γ release assay (IGRA) also left a unique mark in the DNA methylation profile, indicating that DNA methylation-based biomarkers could be used as clinical tools to diagnose tuberculosis infection and assess exposure level (44). Epigenetic modifications are also the basic information for disease differentiation. Although the reference does not directly mention methylation differences in Crohn’s disease (CD) and intestinal tuberculosis (ITB), other studies have found that Mtb infection causes genome-wide changes in chromatin accessibility in host cells; these changes are related to altered gene expression and are enriched for inflammation-related genes, such as those in the type I interferon signalling pathway (45). Therefore, this infection-induced epigenetic reprogramming suggests that there are significant alterations in the pathway of the host immune system in response to pathogens. For older adults and immunocompromised hosts, regulation of the immune response in the induction or suppression of the trained immune response is required. It has been proposed that immunomodulation using trained immunity activators can correct dysfunctions in the innate and adaptive immune systems of older adults, strengthen antimicrobial immunity, and reduce inflammation (11). Therefore, further research and application of this epigenetic reprogramming may offer new strategies for the development of new vaccines (such as those based on the principles of trained immunity) (43), and also provide new directions for precise diagnosis of diseases such as tuberculosis and population-specific immunological interventions.

### 6.2 The dual immunoregulatory role of key transcription factors

A few transcription factors are required for all the links in the immune response to tuberculosis. Although no one has directly studied the CREB3L1 gene in the literature, the dual nature of other transcription factors and signalling pathways in the immunoregulation of tuberculosis has been identified. The induction of the trained immune response is itself the result of a series of transcriptional and epigenetic programmes. For instance, the Mycobacterium tuberculosis secreted protein MPT53 can make pulmonary epithelial cells release TRCR1-rich exosomes, and these exosomes, in turn, drive trained immunity via the TRCR1-FXR2-CLOCK axis to strengthen the host’s defence against M. tuberculosis (43). At this time, CLOCK, an essential transcription factor, and its corresponding histone acetylation are upregulated; at the same time, standard anti-tuberculosis drugs may have adverse effects on the protective immunity of the host. Anti-tuberculosis drugs inhibit the activation and function of immune cells, as shown in the above evidence. Immunological training inducers, such as BCG vaccination and β-glucans, can strengthen the response of innate immune cells by epigenetic and metabolic reprogramming (46). However, Mycobacterium tuberculosis itself may use strategies to counteract or suppress this protective immune training. Studies have shown that, unlike BCG or β-glucan, which reprogram haematopoietic stem cells (HSCs) through type II interferon or IL-1 responses, Mtb infection reprograms HSCs by way of type I interferon (IFN-I) response, thus inhibiting bone marrow haematopoiesis and damaging the development of protective immunological training against Mtb (47). This IFN-I signalling interferes with iron metabolism, causes depolarisation of the mitochondrial membrane potential, and leads to the specific death of myeloid progenitor cells; it is regarded as an immune evasion strategy employed by Mtb to limit bone marrow haematopoiesis and the antimicrobial capacity of the innate immune system (47). This indirectly suggests that, in severe or progressive infections, abnormal activation of certain immune pathways (such as the IFN-I pathway) may be triggered to increase inflammation and fight off the infection, but it can also lead to dysregulation of the immune response and impaired host defence. The formation of trained immunity also involves modifications in the relevant metabolic and transcriptional programmes. For example, HBHA-induced trained immunity has been linked to an increase in the histone marks H3K4me1 and H3K4me3 at the loci of pro-inflammatory cytokine genes (48). In contrast, oxidised low-density lipoprotein (oxLDL)-induced trained immunity depends on the glutamine catabolism pathway (49). Based on the above, it can be seen that transcription factors and epigenetic regulatory networks are involved in regulating the immune response, and therefore, to treat tuberculosis effectively, one should consider modulating the activity of these key transcription factors. Standard therapy may suppress some transcriptional programmes that promote long-term protection (such as Th1/Th17 responses), or fail to correct pathogen-induced deleterious reprogramming; thus, it is not known whether this is related to a higher risk of disease recurrence (47). Therefore, it is proposed that in combination with immunomodulators, anti-tuberculosis therapy can be used to correct harmful immune reprogramming whilst preserving or enhancing protective trained immunity, providing a new theoretical basis for achieving more effective and prolonged treatment.

## 7 Immunomodulatory challenges and strategies in complex comorbidities

### 7.1 Immunometabolic interactions and treatment in diabetes–tuberculosis comorbidity

Diabetes mellitus (DM) and TB are occurring together in an increasing number of cases, and the main reason is that high blood sugar severely weakens the host’s defence mechanism against TB by damaging the immune system; studies have shown that high blood sugar and its products, such as methylglyoxal (MGO), directly inhibit the macrophage’s control of Mtb replication (50). The reduction is partly due to the inhibition of hypoxia-inducible factor-1 (HIF-1)-mediated immunometabolic responses; HIF-1 is a master regulator of macrophage activation and antibacterial function (50), and hyperglycaemia and glycation stress in diabetes suppress HIF-1-regulated gene expression in macrophages, thereby impairing their antibacterial ability (50). Moreover, the immunometabolic disorder of diabetes is bidirectional: diabetes increases the risk of tuberculosis, and active tuberculosis can also impair glycaemic control by inducing insulin resistance and stress-induced hyperglycaemia, thereby forming a vicious circle (51). The immune response in this co-morbidity is characterised by excessive and dysregulated inflammation. Compared with patients with tuberculosis alone, those who have tuberculosis and diabetes mellitus (TB-DM) show a more pronounced systemic inflammatory response, with elevated levels of various pro-inflammatory proteins in the plasma, and this inflammation may continue after the completion of anti-tuberculosis treatment (52). At the local pulmonary level, patients with TB-DM often have impaired pro-inflammatory M1 macrophage polarisation, reduced expression of interferon-γ (IFN-γ) and inducible nitric oxide synthase (iNOS), and increased expression of pro-inflammatory factors such as interleukin-1β (IL-1β); this unbalanced cytokine environment may lead to aggravated immunopathology and poor bacterial control (53). Studies have also found that TB-DM comorbidity is associated with more severe pulmonary radiological lesions and a slow radiological response after treatment; this is linked to persistent low-grade inflammation caused by insufficient upregulation of local anti-inflammatory factors, such as IL-10 (54). Therefore, the treatment strategy for the comorbidity of diabetes mellitus and TB has entered the era of personalised medicine, and the focus of attention is now on HDT with metformin and other antidiabetic drugs. Metformin reduces blood glucose levels and is also known to have immunomodulatory effects; it promotes macrophage autophagy, reduces excessive inflammation, and maintains the metabolic homeostasis of CD8+ T cells. Preclinical research and retrospective clinical studies have both found that it can enhance the efficacy of tuberculosis treatment (55). In terms of future research directions, scientists aim to explore the mechanism by which inflammation is resolved, examine the role of insulin resistance in comorbidity, and develop diagnostic biomarkers for early diagnosis and personalised medicine using artificial intelligence (56).

### 7.2 Dysregulation of the neuroendocrine-immune axis in HIV-TB co-infection

Co-infection of HIV and Mycobacterium tuberculosis (Mtb) is a severe synergistic epidemic, and the biological basis for this phenomenon is the combined damage to the host’s immune system caused by both pathogens. HIV infection severely impairs the cellular immune response required to control Mtb infection by depleting CD4+ T cells, thus significantly increasing the risk of primary tuberculosis or reactivation of latent infection (57). Due to the prolonged presence of this chronic infection and continuous inflammation, the host’s neuroendocrine-immune axis is altered; chronic immune activation stimulates the hypothalamic–pituitary–adrenal (HPA) axis and raises cortisol levels. At the same time, levels of dehydroepiandrosterone (DHEA) and its active metabolites (such as 7-hydroxy-DHEA) decrease, and an unfavourable cortisol/DHEA ratio results (57). Endocrine disorders are related to a weakened immune system and an increase in disease severity. Research in the field of anti-disease strategies has explored how to target the endocrine system to strengthen the immune system, suppress the proliferation of regulatory T cells (Tregs), promote the expansion of Th1-type immune cells, and enhance macrophage-mediated antibacterial responses (57). In addition to the HPA axis, adipokines such as leptin and adiponectin are also dysregulated in HIV-TB co-infection and further impair the energy supply and function of immune cells. Furthermore, this co-infection has reduced diagnostic accuracy, diminished efficacy of anti-tuberculosis and antiretroviral therapy, induced drug toxicity, and elevated the risk of immune reconstitution inflammatory syndrome (IRIS) (57). However, several critical caveats must be emphasised. Clinical evidence for hormonal immunomodulation in TB-HIV is extremely limited. The complex context—ART regimens, drug-drug interactions, IRIS risk—poses substantial challenges. Therefore, neuroendocrine modulation remains highly experimental.

### 7.3 The gut microbiota and derived immunomodulators: a hypothesis-generating perspective

Currently, no direct evidence links B. fragilis PSA, probiotics, or any specific microbial metabolite to improved TB outcomes in humans. The following represents a hypothesis-generating perspective. As the body’s largest immune organ, the gut microbiota helps to regulate the whole-body immune system and inflammation, and new directions for adjuvant treatment of tuberculosis have been proposed. Amongst these, Bacteroides fragilis and its capsular polysaccharide A (PSA) are gut microbiota-derived immunomodulators that have been extensively studied. PSA can be recognised by intestinal immune cells, activates CD4+ T cells, and has the property of bidirectional regulation of inflammation; that is to say, it can suppress inflammation or promote it, which is necessary for controlling infection-related immunopathology. Dysbiosis of the gut microbiota is also closely related to metabolic disorders, such as type 2 diabetes, and diabetes is an independent risk factor for tuberculosis. Research has shown that patients with pulmonary tuberculosis and diabetes (PTB-DM) have reduced α- and β-diversity in their gut microbiota, and a significant decrease in beneficial bacterial groups such as the Spirillaceae and Ruminicoccaceae families has been observed (58). This microbial dysbiosis is associated with changes in faecal and serum metabolomics, including an increase in amino acid (e.g., phenylalanine, tyrosine, tryptophan) metabolic pathways, and it is suggested that the microbiota-metabolite-immune axis may be involved in the pathogenesis of co-morbidities (58). Therefore, probiotic, prebiotic and specific microbial metabolite (such as PSA) interventions aimed at correcting microbiota dysbiosis is theoretically plausible that regulate the host immune response and improve the outcome of tuberculosis. Engineered PSA may be used to develop an effective carbohydrate vaccine adjuvant that enhances the immune response to pathogens such as Mycobacterium tuberculosis (51). Nevertheless, substantial challenges must be overcome before clinical translation: strain specificity, interindividual variability, diet, antibiotic exposure, and reproducibility of microbiome-based interventions all remain major hurdles. We emphasise that microbiome-targeted HDT strategies for TB are currently in the earliest stages of exploration and require rigorous preclinical and clinical validation (**Table 3**).

**Table 3:** Immune characteristics and potential host-directed-therapy targets for tuberculosis patients with major comorbidities.

Comorbidity	Core immune-metabolic abnormalities	Main pathological manifestations	Priority HDT targets	Candidate interventions	Evidence level (in TB Context)
TB-DM	HIF-1 suppression; M1/M2 imbalance; persistent low-grade inflammation	Severe lung lesions; delayed radiological improvement	HIF-1; inflammation resolution; gut microbiota	Metformin; metabolic adjuvants; probiotics	Level B (observational, microbiota) (58); Level C (preclinical metformin) (26); Level A (RCT not stratified for DM) (36); Level E (non-TB) (50)
TB-HIV	CD4+ T-cell depletion; HPA-axis disturbance; cortisol/DHEA imbalance; high IRIS risk	Elevated latent TB reactivation; higher drug toxicity	Neuroendocrine-immune axis; Treg/Th1 balance	DHEA derivatives; immune-modulating adjuvants for IRIS	Level E/F (mechanistic, no clinical evidence; hypothetical) (57)

This table compares immune-metabolic dysregulation and promising HDT targets in TB-DM and TB-HIV co-infection, with evidence levels and key references. Evidence levels: A, RCTs in TB patients; B, observational studies in TB patients; C, preclinical TB animal models; D, *in vitro* TB studies; E, evidence extrapolated from non-TB diseases; F, hypothetical mechanisms. References are numbered according to the main reference list. Abbreviations: DHEA, dehydroepiandrosterone; DM, diabetes mellitus; HDT, host-directed therapy; HIF-1, hypoxia-inducible factor 1; HIV, human immunodeficiency virus; HPA, hypothalamic-pituitary-adrenal; IRIS, immune reconstitution inflammatory syndrome; Mtb, Mycobacterium tuberculosis; TB, tuberculosis; Treg, regulatory T cell.

## 8 Conclusion

Recently, the idea of treatment for pulmonary tuberculosis has been changing, and now it frequently includes concurrent treatment for both the host and the microbe. HDT strategies aim to regulate the immune system and are now extensions of traditional chemotherapy. Their main purpose is to change the host’s immune response and address problems such as immunosuppression, excessive inflammatory damage and the risk of recurrence that occur during chemotherapy alone. Based on expert research, in recent years, many different mechanisms have been identified; specifically, multi-level networks involving immune senescence, immune training, immunometabolic reprogramming, and the neuroendocrine-immune axis have gradually been elucidated. These results have added to our knowledge of the reasons for tuberculosis and, more importantly, provided HDT with an all-encompassing list of targets at the level of epigenetics and systemic physiology.

At present, the application paths for HDT are starting to emerge. From repurposed drugs to new biologics, many candidates for modulating the immune system at a specific level are being developed to either boost defence against bacteria or suppress harmful inflammation for tissue repair. However, the actual problem lies in how to apply this complex knowledge of mechanisms to clinical practice. Given that the immune status of patients with comorbidities of diabetes and HIV infection varies greatly, a single “immune-enhancing” or “anti-inflammatory” approach will not be suitable for all cases. Therefore, the future development will not be the discovery of a ‘panacea’ but the cultivation of precision regulation capabilities. Integrate multi-omics data with clinical information to find biomarkers that can predict how an individual will respond to treatment and their long-term health, thus realising personalised medicine.

The first demand in the future for the application of the theory of High-Definition Therapy is to conduct well-designed clinical trials that verify both its safety and efficacy when combined with standard medical care. Additional research should also move beyond bacterial conversion to explore other hard end points, such as a shortened treatment period, a decrease in recurrence, and improvements in long-term lung function and quality of life. Finally, by combining pathogen-targeted therapy with host-state regulation, a new era of personalised and all-encompassing tuberculosis treatment is hoped to be achieved; this will help curb the spread of tuberculosis and offer a new model for treating other chronic infectious diseases.

Summary of Evidence Strength and Future Directions. Based on our critical assessment:

Currently strongest clinical evidence: None of the reviewed HDT candidates have achieved Level A evidence for routine clinical use in TB. Azithromycin has shown promise in a pilot RCT (Level B) for reducing neutrophilic inflammation, but confirmatory trials are needed.
Moderate evidence (Level B–C): Metformin has been evaluated in one RCT (negative for primary bacteriological endpoint) and several preclinical studies; it may have a role in limiting inflammation but not as a stand-alone microbiological enhancer. Statins show observational evidence for hepatoprotection (Level B) but lack direct immunomodulatory evidence in TB (Level E/F for this indication).
Experimental/Hypothetical (Level E–F): Oxylipin modulation, cholinergic system targeting, microbiome-based therapies, and neuroendocrine interventions remain largely speculative or based on extrapolation from non-TB diseases.
Priority for future research: Well-designed, TB-specific RCTs with patient stratification (based on age, comorbidities, baseline inflammation) are urgently needed. Endpoints should extend beyond sputum conversion to include long-term lung function, recurrence rates, and quality of life.
